# The Interrelationship between Stress, Sugar Consumption and Depression

**DOI:** 10.3390/nu16193389

**Published:** 2024-10-05

**Authors:** Adi Fish-Williamson, Jennifer Hahn-Holbrook

**Affiliations:** Department of Psychology, University of California, Merced, Merced, CA 95343, USA

**Keywords:** diet, added sugar, sugar-sweetened beverages (SSB), chronic stress, depression

## Abstract

Depression is a leading cause of disability in the United States. Previous research has shown that added sugar consumption and stress are both risk factors for depression. Despite evidence that stress predicts added sugar consumption, and both affect the HPA axis, no research has explored how stress, added sugar consumption and depression are related. In this study, we investigated the possible effects of total added sugar and sugar-sweetened beverage consumption on depression, as well as their potential interactions with chronic stress. Measures of sugar consumption, chronic stress and depression were taken in an adult community sample at two time points. We hypothesized that high sugar consumption would predict more depression even after stress was statistically adjusted for, but that stress would moderate the relationship between added sugar consumption and depressive symptoms, amplifying the effect. We found that both total sugar consumption and sugar-sweetened beverage consumption at baseline predicted depressive symptoms one month later. However, only sugar-sweetened beverage consumption was a significant predictor of depression after controlling for stress, possibly because stress is related to diet quality. Stress did not moderate the relationship between added sugar consumption and depressive symptoms. These results suggest that stress should be included in future research on sugar and depression.

## 1. Introduction

Depression is a serious public health concern globally. One in ten people in the United States suffer from depression [1]. In adolescents and young adults, the prevalence is closer to 1 in 5 [1], making it the most common mental health problem for this age group [2]. Depression can be a physically and emotionally debilitating disorder, and it significantly increases the risk of cardiovascular disease, stroke, diabetes, obesity and suicide [3,4].

Like many disorders, the true cause of depression is unknown, but there is strong evidence for both genetic and environmental risk factors [5,6]. Research investigating lifestyle factors influencing depression has been expanding and is of particular interest due to the potential modifiability of daily habits. Alongside adequate sleep and exercise, diet has been pinpointed as a predictor of depression [6,7]. Various dietary factors have been shown to increase depression risk, such as consumption of processed foods and fast food [8,9]. Processed added sugar consumption in particular has been identified as a predictor of depressive symptoms [8,9,10]. Given that added sugar is one of the largest sources of calories in the United States [11], research has investigated whether it plays a role in mental illness.

### 1.1. Sugar and Depression

High added sugar consumption can adversely affect physical health and lead to obesity, type II diabetes, cardiovascular disease, and mortality [12,13,14]. Interest in the relationship between added sugar consumption and mental health has been growing, and while the field is still nascent, multiple studies have investigated the relationship between added sugar consumption and depression [10,15,16,17]. For example, in a cross-sectional study of 74 women, Fish and George (2020) found a positive association between sugar consumption and depressive symptoms. Moreover, in a longitudinal study of 15,546 Spanish adults in the SUN cohort, Sanchez-Villegas and colleagues (2018) found that those in the highest quartile of added sugar consumption at the 10 year follow-up were at a significantly higher risk for depression [17]. In the Whitehall II study of 23,245 person-observations, it was found that men with the highest quartile of sugar consumption had a 25% increase in odds of having a common mental health disorder after 5 years [16]. This effect was independent of other dietary, socioeconomic and health factors. In a meta-analysis of observational studies examining sugar-sweetened beverage consumption and depression risk, 6 out of 10 of the articles found a statistically significant relationship [15]. Overall, the meta-analysis concluded that the combined risk of depression for people consuming the highest (versus lowest) consumption of SSBs was 1.31, although there was significant heterogeneity in effect sizes across studies. Together, these studies suggest that high added sugar consumption may contribute to adverse mental health outcomes [17]. One key limitation of the literature on sugar consumption and depression, however, is that fact that all studies, to our knowledge, have been correlational. Thus, the possibilities of reverse causality (e.g., that depression causes more sugar consumption) or that some third variable could be leading to a spurious association between sugar consumption and depression are left open. One potential third variable that could explain the relationship between sugar consumption and depression is stress, as it has been linked to both sugar consumption and depression. One goal of the current study is to see whether the relationship between sugar consumption and depression persists after statistically adjusting for stress; we hypothesized that this relationship would persist.

### 1.2. Stress and Depression

Decades of research have established a robust relationship between life stress and depression [18,19]. A meta-analysis of 62 articles and 44,066 participants found that people who experienced early-life stress were more likely to develop major depressive disorder before the age of 18 than those without a history of early-life stress [20]. In addition to early-life stress, various types of stress, such as chronic psychosocial stress, ongoing stress and post-traumatic stress, increase depression risk [21,22,23]. It is not just stress that is encountered in adulthood; early-life stress can affect the HPA axis and depression risk as well, for example, adverse childhood experiences (ACEs) [24]. People with ACEs have a higher risk of developing depression [24]. Severe trauma essentially rewires the HPA response in a way that promotes a chronically activated stress response [25]. Stress not only increases depression risk, but it also is associated with higher consumption of sugar.

### 1.3. Stress and Sugar Consumption

Stress is associated with altered eating behavior, such as more frequent consumption of sweets and fast food and less frequent consumption of fruits and vegetables [26]. These findings are, in part, due to comfort eating, a behavioral coping response [27]. When faced with similar levels of stress, comfort eaters experienced reduced perceived stress compared with those who did not engage in comfort eating [27]. The relationship between stress and eating is complexified by the addictive nature of comfort foods such as those with high sugar content. Stress is a key factor in the development of addiction. Uncontrolled stress changes eating patterns by increasing hyperpalatable food consumption (e.g., comfort foods high in sugar and fat), which can increase allostatic load and metabolic disease over time [28]. This is because food and some drugs exploit similar pathways in the brain by engaging the dopaminergic system [29]. Subsequently, a cyclical relationship emerges that begins with stress leading to comfort eating, which leads to obesity, metabolic disease and, ultimately, increasing stress [30]. Building on our understanding of the relationship between stress and health outcomes, the current study aimed to explore the complex interplay between added sugar consumption, stress and depressive symptoms. Critically, there is empirical evidence to suggest that chronic stress may interact with sugar consumption to shape biological risk factors for depression.

Specifically, a landmark study by von Dawans and colleagues (2021) found that people who had high glucose in their system showed a greater blood sugar increase in response to stress than people who had low glucose in their system before a stressor [31]. This effect was driven by the stress-induced decrease in insulin, which helps to capture and store excess glucose in the blood stream [31]. In an earlier study that examined how cortisol and epinephrine affect how the body handles sugar, healthy participants received either cortisol, epinephrine or saline infusions before and after consuming glucose. Aligning with the previous study, the findings showed that blood sugar spiked more after the cortisol infusion and epinephrine infusions than in the saline control group [32]. The relationship between blood sugar and stress hormones has also been examined in non-human animals. In dogs, for example, glucagon, epinephrine and cortisol were found to work together to rapidly increase blood sugar, particularly when all three were present [33]. Although these are lab studies examining short-term effects, it is plausible these findings have long-term health implications. In a study of 251 people with acute coronary syndromes, higher levels of cortisol were linked to more severe coronary events and elevated blood sugar [34]. Given that insulin resistance and chronically high blood sugar are well-established risk factors for depression [35], a reexamination of the relationship between sugar consumption and depression that explores the potential interaction effect of chronic stress is warranted. We hypothesized that chronic stress and sugar consumption could biologically interact to predict depressive symptoms by amplifying the effects of added sugar consumption on depression through high blood glucose and insulin resistance.

### 1.4. The Current Study

The goal of the current study was to investigate the potentially complex relationship between added sugar consumption, stress and depression. Our aims were three-fold. First, we wanted to try and replicate results of previous studies that have found a positive correlation between sugar consumption and depression. Secondly, we wanted to establish whether stress was related to sugar consumption and, further, to rule out the possibility that stress acts as a third variable leading to spurious correlations between sugar consumption and depression. Finally, we aimed to test the novel hypothesis that stress can interact with sugar consumption and depression risk in adults, amplifying the deleterious effects of sugar consumption on mood. Specifically, we predicted that people who eat diets high in sugar and experience higher levels of chronic stress would report high scores on a scale measuring depressive symptoms than those who consume high levels of sugar or experience high levels of stress alone. To test this interaction model, we recruited US adults to complete two online surveys. The survey included questionnaires for diet, stress, and depressive symptoms.

## 2. Methods

### 2.1. Participants and Data Collection

Adults over the age of 18 from the US, UK, Australia and New Zealand were invited to participate in an online survey on the effects of diet and stress on wellbeing, and the investigators repeated the survey one month later. Surveys were offered in English in February and March 2024. Participants were recruited through Prolific. Adults were eligible if they were over the age of 18; lived in the United States, the United Kingdom, Australia or New Zealand; and spoke English. Prolific participants received a payment of USD 2 upon completion of the survey at baseline and USD 3 upon completion of the survey at the follow-up, 1 month later. The survey took approximately 10 min to complete at each time point. To make sure all participants were humans (not bots), the study included one short-answer question asking participants to speculate what the study was about. Participants who failed to answer the question appropriately were removed from analysis. Given that this was the first study to examine the interaction of sugar consumption, stress and depression, a conventional power analysis using effect sizes from previous findings was not possible. Therefore, in line with the commonly used recommendation by Brysbaert (2019) for detecting clinically significant results, we aimed to collect a sample of 250 participants. This number was picked in anticipation that 15% of the data may not be useable due to incomplete responses or participant inattention. This would leave a final sample of 212 participants, giving us over >80% power to detect and the recommended interaction effect size of Cohen’s D of 0.40 [36]. Ultimately, a total of 250 participants completed data collection at baseline. A total of 3 participants were removed for failing the attention check question, and 29 were removed for not participating in the second survey; 1 participant that successfully completed the first wave timed out during the follow-up survey, resulting in 217 participants included in the study.

### 2.2. Depressive Symptoms

Depressive symptomology were assessed via the brief version of the Center for Epidemiologic Studies—Depression scale (CES-D), a self-report scale designed to measure depressive symptoms in the general population, with higher scores indicating higher levels of depressive symptoms [37]. We used the brief 20-item version of the CES-D to minimize participant burden. The Cronbach’s Alpha for the CES-D was α = 0.943. We planned to conduct analysis continuously to capture the degree of depressive symptoms.

### 2.3. Measures of Stress

The assessment of stress was made by administering two chronic stress scales. The Adverse Childhood Experiences (ACEs) scale was used to assess the degree to which an individual had experienced childhood trauma [38]. Participants responded to 14 items on a dichotomous scale, and scores were obtained by assigning each item a point if present (1) and none if absent (0). Items were then summed into an ACEs index that ranged from 0 to 14, with higher scores indicating more adverse childhood experiences [38]. In addition, chronic stress was measured using the 9-item Trier Inventory for Chronic Stress (TICS-EN-9), which uses a five-point scale to assess how often participants have encountered specific chronic-related stress situations or had specific experiences within the previous 3 months. Results were scored on a five-point scale (0 = never, 1 = rarely, 2 = sometimes, 3 = often, 4 = very often) [39]. When used together, these scales encompass both chronic stress resulting from childhood trauma and current chronic life stress, providing a more comprehensive assessment of chronic stress.

### 2.4. Measures of Added Sugar Consumption

Participants’ dietary consumption was measured using items from the food frequency questionnaire of the Growing Up in New Zealand study (*GUiNZ*) [40,41]. Participants answered questions on how many servings of each dietary item they ate or drank over the past 4 weeks. The food items used to reflect sugar consumption were: ice cream; cakes or biscuits/cookies; soft drinks or energy drinks excluding diet drinks, fruit juices, flavored waters, and sports waters; confectionary; lollies/lollipops/suckers; sweets; and chocolates. We created a composite variable using all the items containing added sugar to measure total sugar consumption (M = 4.49, SD = 1.49), and also created a separate variable to measure SSB consumption specifically (M = 3.53, SD = 2.16).

### 2.5. Covariates

The following covariates were included in the model given that previous studies have shown they are related to depressive symptoms, stress and/or sugar consumption: age, household income, education, sex, fruit consumption, and vegetable consumption.

### 2.6. Ethical Approval

The University of California, Merced Institutional Review Board, holding Department of Health and Human Services Federalwide Assurance #00005105, reviewed and determined the protocol of the current study to be exempt according to Exempt Category 2, which states: (i). Research that only includes interaction involving educational tests (cognitive, diagnostic, aptitude, achievement, survey procedures, interview procedures or observation of public behavior including visual or auditory recording) if at least one of the following criteria is met: The information obtained is recorded by the investigator in such a manner that the identity of the human subjects cannot be ready ascertained, either directly or through identifiers linked to the subject.

## 3. Statistical Analysis

We used stepwise linear regressions in SPSS version 29, IBM, Chicago, USA to assess if the total sugar consumption and SSB consumption at baseline predicted depressive symptoms at the 1-month follow-up in separate models. We chose this stepwise approach to allow for the examination of sugar alone and then sugar controlling for stress. We attempted to prevent potential issues that can arise in stepwise linear regressions, such as overfitting and multicollinearity, by limiting the analysis to only include variables that were supported by previous research. Step one included each sugar variable alone, step 2 included the TICS and ACEs, step 3 included fruit and vegetable consumption and, lastly, participant demographics were added in step 4. Separately, we tested if the TICS and ACEs scores interacted with sugar consumption to predict depressive symptoms. For the total sugar model, we included an interaction variable created by separately multiplying the TICS and ACEs scores by the total sugar consumption composite variable. For the SSB model, the interaction variables were created by multiplying the TICS and ACEs score variables by the SSB consumption variable. All variables were mean-centered before analysis to help with model interpretation. *p* values (<0.05) and 95% confidence intervals (CI) that did not overlap with zero were be used to examine whether effects were significant.

## 4. Results

### 4.1. Demographics

A total of 217 participants completed both data collection waves and were included in this study. Of these participants, 66.8% identified as female, 84.8% resided in the UK, 84.3% were White and the average age was 60.24 (SD = 11.53, RANGE = 40–93). See Table 1 for the complete demographic information of the sample. 91 people (41.9%) met the CES-D cutoff score for possible depression (i.e., score of 20 or more) [42], with an overall mean CES-D score of 19.456. The median frequency of total sugar consumption fell between 1–2 times per week and 3–4 times per week, and the mean frequency of SSB consumption was between 2–3 times per month and 1–2 times per month. See Table 2 for a correlation matrix showing how demographics, sugar consumption, stress and depression related to each other.

### 4.2. Concurrent Analysis

In the total added sugar model, baseline sugar consumption predicted baseline depressive symptoms (β = 0.186, *p* < 0.006). The interaction variables were not significant predictors of depressive symptoms. No other significant predictors emerged.

### 4.3. Does Total Sugar Consumption at Baseline Predict Depression at Follow-Up?

As is consistent with previous research, people who consumed more total added sugar at baseline reported more depressive symptoms 1 month later than those who consumed less added sugar at baseline (see Table 3 for the full stepwise regression model). This effect was no longer significant when the stress scales were included in the model, or when fruits and vegetables consumption and demographic variables were included as covariates.

### 4.4. Does SSB Consumption at Baseline Predict Depression at Follow-Up?

SSB consumption at baseline predicted more depressive symptoms at the 1-month follow-up, an effect that remained significant when the stress scales, fruit and vegetable consumption and demographics were added into the model.

### 4.5. Does Chronic Stress Predict Added Sugar Consumption?

TICS scores at baseline predicted total sugar consumption (β = 0.252, *p* < 0.001) and SSB consumption (β = 0.160, *p* < 0.034) at follow-up. ACEs scores did not predict sugar consumption (total sugar consumption: β = 0.128, *p* = 0.085, SSB consumption: β = 0.069, *p* = 0.357).

### 4.6. Do Sugar Consumption and Stress Interact?

The interaction between total sugar consumption and chronic stress at baseline did not significantly predict CES-D scores at the follow-up in any of steps that we examined (total sugar consumption and TICS: β = −0.051, *p* = 0.36, total sugar consumption and ACEs: β = −0.019, *p* = 0.797). Similarly, the interaction between SSB consumption and chronic stress at baseline did not significantly predict CES-D scores at the follow-up (SSB consumption and TICS: β = −0.079, *p* = 0.55, SSB consumption and ACEs: β = 0.022, *p* = 0.708).

### 4.7. Does Depression at Baseline Predict Sugar Consumption at Follow-Up?

We also wanted to examine the possibility of reverse causality—the idea that depression causes an increase in sugar consumption and that is why there is a positive correlation between sugar consumption and depression. There was a trend where higher CES-D scores at baseline predicted SSB consumption at follow-up (β = 0.118, *p* = 0.085). However, this result became nonsignificant when the TICS and ACEs scores at baseline were added to the model (β = 0.060, *p* = 0.562). This null result did not change when other nutritional variables or demographics variables were added to the model. Additionally, controlling for SSB at baseline did not change the results.

In the total added sugar consumption model, depression at baseline significantly predicted higher sugar consumption at follow-up (β = 0.136, *p* = 0.047). When stress was included in the model, this effect was no longer significant (β = −0.001, *p* = 0.990). Including other dietary factors and demographics did not change this pattern of results. Adding total sugar consumption at baseline to the model resulted in a trend where higher levels of depression at baseline predicted less sugar consumption at follow-up (β = −0.133, *p* = 0.061).

### 4.8. Post Hoc Test Examining If Sugar Mediates the Relationship between Stress and Depression

We conducted exploratory post hoc analysis using the process macro in SPSS to see if stress predicted higher sugar consumption, which, in turn, predicted more depression. In the total sugar consumption model and the SSB consumption model, basic conditions of mediation were not met. Specifically, although stress did predict higher total sugar consumption (β = 0.102, *p* = 0.018), when stress and total sugar were included in the model together, total sugar was no longer a significant predictor of depression (β = 0.051, *p* = 0.520). In addition, in the SSB model, stress did not predict SSB consumption (β = 0.026, *p* = 0.704). Moreover, the indirect effects in both models were not statistically significant (SSB: 95% CI [−0.0182, 0.0304]), total sugar: 95% CI [−0.019, 0.0311]).

## 5. Discussion

The goals of the current study were to examine if total sugar consumption and SSB consumption predicted depressive symptoms in the general population and to investigate whether added sugar consumption and chronic stress interact to increase depression risk. This study found that both total sugar consumption and SSB consumption at baseline predicted depressive symptoms one month later; however, after controlling for stress, only SSB consumption remained a significant predictor of depression. Although the relationship between total sugar consumption and depression seemed to be explained by stress, the relationship between SSB consumption and depressive symptoms persisted even after controlling for multiple covariates such as stress levels, other dietary factors and demographics. Our SSB results are in line with existing research. In a previous SSB consumption and depression meta-analysis, 6 of the 10 studies included found a positive relationship between SSB consumption and depressive symptoms [15]. It is important to note that all of the current studies, including our own, were correlational in nature.

While there is strong evidence to suggest that sugar may increase depression risk through biological mechanisms such as inflammation, the gut–brain axis and disrupted HPA function [43,44,45], other research indicates that mental states like depression may influence eating behavior and lead to an increase in ingestion of comfort foods, including those with added sugar [27]. While our post hoc analyses did find that when people are stressed, they eat more sugar, and when people eat more sugar, they are more likely to be depressed, total added sugar consumption was not a true mediator in the relationship between stress levels and depressive symptoms. Thus, our findings may reflect SSB consumption leading to depression or depression leading to SSB consumption. There is also the possibility that a third variable accounts for this relationship. Our results suggest that stress does act as a third variable in the relationship between total sugar consumption and depression, leading to a spurious correlation that becomes nonsignificant when stress is added to the model. Therefore, we encourage future researchers to take stress into account when examining the relationship between sugar consumption and depression. Previous studies have included socioeconomic factors, health behaviors and existing health conditions as covariates [46,47,48]. However, that is the extent of variables that are typically considered; therefore, exploratory studies on the subject that include psychological stress and eating behaviors are warranted.

The null findings for the interaction variables were inconsistent with our hypothesis based on previous research [31] that found that people who had high glucose in their system experienced the strongest physiological reactions to stress. Given these findings, we hypothesized that the long-term combination of stress and added sugar consumption would interact to predict depression. A key difference is that our study measured psychological stress with an outcome of depression, whereas Von Dawans et al. (2021) [31] focused on the effect of glucose consumption on physiological stress. While it is possible that sugar and chronic stress do not interact to predict depressive symptoms, it is also possible that the interaction exists, but was not captured by self-reported measures of chronic psychological stress. Additionally, the study by Von Dawans and team (2021) [31] was designed to assess an acute stress reaction, whereas the current study is looking at chronic stress. While both acute and chronic stress involve an HPA axis response, and therefore theoretically trigger the release of stress hormones such as cortisol, the long-term activation of the HPA axis may affect sugar metabolism differently than sudden activation by an acute stressor.

### 5.1. Limitations

The results of this study should be considered in the context of several limitations. As mentioned previously, the short interval between the baseline and one-month follow-up did not allow for meaningful variability in depression or sugar consumption levels. Specifically, due to the high correlation between depression levels at baseline and at the 1-month follow-up (Pearson’s *r* = 0.846), we were unable examine if sugar consumption at baseline predicts changes in depression over time. Longitudinal studies that include multiple measures of both sugar consumption and depressive symptoms over years are needed to untangle the directionality of this relationship. We used Prolific to recruit for this study, which, like any online recruitment platform, introduces the potential for bots and participant distraction during the survey compared to a lab study [49]. Additionally, our cohort primarily consisted of white women in the UK with above-average levels of depression (41.9% met the CES-D cutoff for possible depression), limiting the generalizability of our findings to other populations. Women are more likely to report depressive symptoms, which may have impacted the rates of depression in the current study (Kessler, 2003) [50]. It is also possible that we had such high rates of depression because the advertising for the study included a mention of depression. Given that diets vary widely cross-culturally, it is possible that our findings would have been different if the sample reflected a more diverse population. Furthermore, the use of a self-report screening tool to measure depressive symptoms may not accurately capture clinical levels of depression. Although the CES-D is regarded as reliable, clinical interviews are the gold standard for assessing depressive disorders [37]. Additionally, the current study measured added sugar consumption with a food frequency questionnaire instead of the gold-standard method of collecting dietary data via multiple dietary recalls conducted by a trained interviewer, which may lead to recall bias or underreporting of sugar consumption. Lastly, it is possible that we did not find an interaction effect because there was not enough power to detect it.

### 5.2. Future Directions

Future research should incorporate physiological stress measures in studies examining sugar and depression and consider longitudinal designs with longer follow-up periods. Although randomized control trials are the gold standard, a trial assessing sugar, stress and depressive symptoms in humans would be challenging. An alternative approach could be to conduct a sugar-free intervention in people with depression and include physiological stress and depressive symptoms that are measured before, during and after the intervention. In this type of design, it would be possible to assess the directionality of the relationship and identify potential interactions. Incorporating biological variables such as genetic information to either type of design in future studies would be valuable, given that certain genes influence the stress response and metabolism [5,51].

## 6. Conclusions

This study suggests that psychological stress does not interact with sugar consumption to predict depressive symptoms. However, we did find evidence that sugar and depression are positively correlated even after statistically adjusting for stress. Understanding whether added sugar consumption is a biological risk factor for depression is crucial. If so, it could serve as a target for interventions preventing a disorder that affects millions of people in the US alone.

## Figures and Tables

**Table 1:** Participant demographics.

Demographic Variables	*N*	M (SD)/%
Age	215	60.24 (11.531)
Sex	217	
Male		33.20%
Female		66.80%
Race/ethnicity	217	
White		84.30%
Black or African		4.60%
Indigenous or Native		5.10%
Asian		0.50%
Pacific Islander		1.40%
Hispanic or Latino/a		3.20%
Other		0.90%
Previous chronic condition	217	
Yes		71.00%
No		29.00%
Country of residence	217	
USA		9.20%
UK		84.80%
New Zealand		0.90%
Australia		5.10%
Education	217	
No formal education		0.50%
Some primary education		2.30%
Graduated high school		17.10%
Some post-secondary education		17.10%
Completed post-secondary education		5.50%
Bachelor’s degree		39.20%
Master’s degree		15.20%
Doctorate		3.20%
Number of people in household	217	3.03 (1.475)
Combined household income	217	
USA	20	$40,001–$60,000
UK	184	£45,001–£60,000
New Zealand	11	AUD 75,001–95,000
Australia	2	NZD 110,001–130,000
CES-D score	217	19.456 (12.741)
TICS score	217	15.475 (6.745)
ACEs score	217	2.65 (2.054)
Total sugar consumption SSB consumption	217 217	4.487 (1.491) 13.461 (4.474)

**Table 2:** Correlation matrix of PPD, stress and sugar consumption over time.

Variables	1	2	3	4	5	6	7	8	9	10	11	12	13
1. CES-D scores at baseline	1	0.846 **	0.747 **	0.635 **	0.383 **	0.191 **	0.142 *	0.174 *	0.119	−0.152	0.008	−0.070	−0.196
2. CES-D scores at follow-up	-	1	0.687 **	0.756 **	0.359 **	0.142 *	0.167 *	0.169 *	0.140 *	−0.157 *	−0.015	−0.018	−0.191 **
3. TICS scores at baseline	-	-	1	0.763 **	0.429 **	0.161 *	0.197 **	0.026	0.130	−0.170 *	0.079	0.060	−0.117
4. TICS scores at follow up	-	-	-	1	0.322 **	0.176 **	0.242 **	0.101	0.174 *	−0.126	0.079	0.093	−0.142 *
5. ACEs scores	-	-	-	-	1	0.001	−0.019	−0.008	−0.001	−0.047	0.080	0.007	−0.164 *
6. Total sugar consumption at baseline	-	-	-	-	-	1	0.753 **	0.623 **	0.447 **	−0.181 *	−0.063	0.001	0.077
7. Total sugar consumption at follow up	-	-	-	-	-	-	1	0.390 **	0.616 **	−0.160 *	−0.054	0.009	0.059
8. SSB consumption at baseline	-	-	-	-	-	-	-	1	0.541 **	−0.227 **	−0.185 **	−0.083	0.048
9. SSB consumption at follow-up	-	-	-	-	-	-	-	-	1	−0.241 **	−0.118 **	−0.123	0.001
10. Age	-	-	-	-	-	-	-	-	-	1	0.126	0.034	−0.058
11. Sex	-	-	-	-	-	-	-	-	-	-	1	0.114	−0.025
12. Education	-	-	-	-	-	-	-	-	-	-	-	1	0.146 *
13. Income	-	-	-	-	-	-	-	-	-	-	-	-	1

* Correlation is significant at the 0.05 level (2-tailed); ** Correlation is significant at the 0.01 level (2-tailed).

**Table 3:** Stepwise regressions of sugar consumption at baseline as a predictor of depressive symptoms at 1 month follow-up.

Total Sugar Consumption				SSB Consumption	
Step 1: Sugar Predicting Depressive Symptoms					
	Stand. B	*p*-value		Stand. B	*p*-value
Total Sugar Consumption	0.161	0.018	SSB Consumption	0.163	0.017
Step 2: Time 1 Stress and Interactions					
Total Sugar Consumption	0.039	0.448	SSB Consumption	0.15	0.002
TICS Score	0.642	<0.001	TICS Score	0.647	<0.001
ACEs Score	−0.084	0.132	ACEs Score	−0.082	0.129
Step 3: Dietary Factors Added to the Model					
	Stand. B	*p*-value		Stand. B	*p*-value
Total Sugar	0.04	0.435	SSB Consumption	0.147	0.003
TICS Score	0.641	<0.001	TICS Score	0.646	<0.001
ACEs Score	−0.081	0.146	ACEs Score	−0.08	0.141
Total Fruit	0.042	0.46	Total Fruit	0.031	0.578
Total Vegetables	−0.048	0.4	Total Vegetables	−0.038	0.497
Step 4: Demographics Added to the Model					
	Stand. B	*p*-value		Stand. B	*p*-value
Total Sugar	0.04	0.438	SSB Consumption	0.142	0.006
TICS Score	0.644	<0.001	TICS Score	0.649	<0.001
ACEs Score	−0.072	0.196	ACEs Score	−0.069	0.208
Total Fruit	0.048	0.4	Total Fruit	0.034	0.546
Total Vegetables	−0.009	0.873	Total Vegetables	−0.003	0.965
Age	−0.016	0.763	Age	0	0.995
Sex	−0.064	0.211	Sex	−0.044	0.388
Education	−0.052	0.338	Education	−0.039	0.464
Income	−0.095	0.071	Income	−0.101	0.049

## Data Availability

Data available on request.

## References

[1] Goodwin R.D., Dierker L.C., Wu M., Galea S., Hoven C.W., Weinberger A.H. (2022). Trends in U.S. Depression Prevalence from 2015 to 2020: The Widening Treatment Gap. Am. J. Prev. Med..

[2] James S.L., Abate D., Abate K.H., Abay S.M., Abbafati C., Abbasi N., Abbastabar H., Abd-Allah F., Abdela J., Abdelalim A. (2018). Global, Regional, and National Incidence, Prevalence, and Years Lived with Disability for 354 Diseases and Injuries for 195 Countries and Territories, 1990–2017: A Systematic Analysis for the Global Burden of Disease Study 2017. Lancet.

[3] Penninx B.W., Milaneschi Y., Lamers F., Vogelzangs N. (2013). Understanding the Somatic Consequences of Depression: Biological Mechanisms and the Role of Depression Symptom Profile. BMC Med..

[4] Rihmer Z. (2007). Suicide Risk in Mood Disorders. Curr. Opin. Psychiatry.

[5] Caspi A., Sugden K., Moffitt T.E., Taylor A., Craig I.W., Harrington H., McClay J., Mill J., Martin J., Braithwaite A. (2003). Influence of Life Stress on Depression: Moderation by a Polymorphism in the 5-HTT Gene. Science.

[6] Lopresti A.L., Hood S.D., Drummond P.D. (2013). A Review of Lifestyle Factors That Contribute to Important Pathways Associated with Major Depression: Diet, Sleep and Exercise. J. Affect. Disord..

[7] Lassale C., Batty D., Baghdadli A., Jacka F., Sanchez-Villegas A., Kivimäki M., Akbaraly T. (2018). Healthy Dietary Indices and Risk of Depressive Outcomes: A Systematic Review and Meta-Analysis of Observational Studies: Molecular Psychiatry. Mol. Psychiatry.

[8] Pagliai G., Dinu M., Madarena M.P., Bonaccio M., Iacoviello L., Sofi F. (2021). Consumption of Ultra-Processed Foods and Health Status: A Systematic Review and Meta-Analysis. Br. J. Nutr..

[9] Sánchez-Villegas A., Toledo E., De Irala J., Ruiz-Canela M., Pla-Vidal J., Martínez-González M.A. (2012). Fast-Food and Commercial Baked Goods Consumption and the Risk of Depression. Public Health Nutr..

[10] Fish A., George G. (2020). Added Sugar Consumption and the Relationship with Mood Disorders in Women. J. Fam. Consum. Sci..

[11] U.S. Department of Health & Human Services (2020). 2015–2020 Dietary Guidelines for Americans.

[12] Alexander Bentley R., Ruck D.J., Fouts H.N. (2020). U.S. Obesity as Delayed Effect of Excess Sugar. Econ. Hum. Biol..

[13] Collin L.J., Judd S., Safford M., Vaccarino V., Welsh J.A. (2019). Association of Sugary Beverage Consumption with Mortality Risk in US Adults: A Secondary Analysis of Data from the REGARDS Study. JAMA Netw. Open.

[14] Taskinen M.-R., Packard C.J., Borén J. (2019). Dietary Fructose and the Metabolic Syndrome. Nutrients.

[15] Hu D., Cheng L., Jiang W. (2019). Sugar-Sweetened Beverages Consumption and the Risk of Depression: A Meta-Analysis of Observational Studies. J. Affect. Disord..

[16] Knüppel A., Shipley M.J., Llewellyn C.H., Brunner E.J. (2017). Sugar Intake from Sweet Food and Beverages, Common Mental Disorder and Depression: Prospective Findings from the Whitehall II Study. Sci. Rep..

[17] Sanchez-Villegas A., Zazpe I., Santiago S., Perez-Cornago A., Martinez-Gonzalez M.A., Lahortiga-Ramos F. (2018). Added Sugars and Sugar-Sweetened Beverage Consumption, Dietary Carbohydrate Index and Depression Risk in the Seguimiento Universidad de Navarra (SUN) Project. Br. J. Nutr..

[18] Hammen C. (2005). Stress and Depression. Annu. Rev. Clin. Psychol..

[19] Kessler R.C. (1997). The Effects of Stressful Life Events on Depression. Annu. Rev. Psychol..

[20] LeMoult J., Humphreys K.L., Tracy A., Hoffmeister J.-A., Ip E., Gotlib I.H. (2020). Meta-Analysis: Exposure to Early Life Stress and Risk for Depression in Childhood and Adolescence. J. Am. Acad. Child Adolesc. Psychiatry.

[21] Charney D.S., Manji H.K. (2004). Life Stress, Genes, and Depression: Multiple Pathways Lead to Increased Risk and New Opportunities for Intervention. Sci. STKE.

[22] Morrill E.F., Brewer N.T., O’Neill S.C., Lillie S.E., Dees E.C., Carey L.A., Rimer B.K. (2008). The Interaction of Post-Traumatic Growth and Post-Traumatic Stress Symptoms in Predicting Depressive Symptoms and Quality of Life. Psychooncology.

[23] Siegrist J. (2008). Chronic Psychosocial Stress at Work and Risk of Depression: Evidence from Prospective Studies. Eur. Arch. Psychiatry Clin. Neurosci..

[24] Heim C., Newport D.J., Mletzko T., Miller A.H., Nemeroff C.B. (2008). The Link between Childhood Trauma and Depression: Insights from HPA Axis Studies in Humans. Psychoneuroendocrinology.

[25] Iob E., Baldwin J.R., Plomin R., Steptoe A. (2021). Adverse Childhood Experiences, Daytime Salivary Cortisol, and Depressive Symptoms in Early Adulthood: A Longitudinal Genetically Informed Twin Study. Transl. Psychiatry.

[26] Mikolajczyk R.T., El Ansari W., Maxwell A.E. (2009). Food Consumption Frequency and Perceived Stress and Depressive Symptoms among Students in Three European Countries. Nutr. J..

[27] Finch L.E., Tomiyama A.J. (2015). Comfort Eating, Psychological Stress, and Depressive Symptoms in Young Adult Women. Appetite.

[28] Yau Y.H.C., Potenza M.N. (2013). Stress and Eating Behaviors. Minerva Endocrinol..

[29] Lustig R.H. (2010). Fructose: Metabolic, Hedonic, and Societal Parallels with Ethanol. J. Am. Diet. Assoc..

[30] Tomiyama A.J. (2019). Stress and Obesity. Annu. Rev. Psychol..

[31] Von Dawans B., Zimmer P., Domes G. (2021). Effects of Glucose Intake on Stress Reactivity in Young, Healthy Men. Psychoneuroendocrinology.

[32] Waldhäusl W.K., Gasic S., Bratusch-Marrain P., Komjati M., Korn A. (1987). Effect of Stress Hormones on Splanchnic Substrate and Insulin Disposal after Glucose Ingestion in Healthy Humans. Diabetes.

[33] Eigler N., Saccà L., Sherwin R.S. (1979). Synergistic Interactions of Physiologic Increments of Glucagon, Epinephrine, and Cortisol in the Dog: A Model for Stress-Induced Hyperglycemia. J. Clin. Investig..

[34] Stubbs P.J., Laycock J., Alaghband-Zadeh J., Carter G., Noble M.I.M. (1999). Circulating Stress Hormone and Insulin Concentrations in Acute Coronary Syndromes: Identification of Insulin Resistance on Admission. Clin. Sci..

[35] Kan C., Silva N., Golden S.H., Rajala U., Timonen M., Stahl D., Ismail K. (2013). A Systematic Review and Meta-Analysis of the Association between Depression and Insulin Resistance. Diabetes Care.

[36] Brysbaert M. (2019). How Many Participants Do We Have to Include in Properly Powered Experiments? A Tutorial of Power Analysis with Reference Tables—PMC. J. Cogn..

[37] Radloff L.S. (1977). The CES-D Scale: A Self-Report Depression Scale for Research in the General Population. Appl. Psychol. Meas..

[38] Finkelhor D., Shattuck A., Turner H., Hamby S. (2015). A Revised Inventory of Adverse Childhood Experiences. Child Abuse Negl..

[39] Petrowski K., Kliem S., Albani C., Hinz A., Brähler E. (2019). Norm Values and Psychometric Properties of the Short Version of the Trier Inventory for Chronic Stress (TICS) in a Representative German Sample. PLoS ONE.

[40] Growing Up Reports: Growing Up in New Zealand. https://www.growingup.co.nz/growing-reports.

[41] Gontijo De Castro T., Lovell A., Santos L.P., Jones B., Wall C. (2023). Maternal Determinants of Dietary Patterns in Infancy and Early Childhood in the Growing up in New Zealand Cohort. Sci. Rep..

[42] Jiang L., Wang Y., Zhang Y., Li R., Wu H., Li C., Wu Y., Tao Q. (2019). The Reliability and Validity of the Center for Epidemiologic Studies Depression Scale (CES-D) for Chinese University Students. Front. Psychiatry.

[43] Eisenberger N.I., Inagaki T.K., Mashal N.M., Irwin M.R. (2010). Inflammation and Social Experience: An Inflammatory Challenge Induces Feelings of Social Disconnection in Addition to Depressed Mood. Brain Behav. Immun..

[44] Harrell C.S., Burgado J., Kelly S.D., Johnson Z.P., Neigh G.N. (2015). High-Fructose Diet during Periadolescent Development Increases Depressive-like Behavior and Remodels the Hypothalamic Transcriptome in Male Rats. Psychoneuroendocrinology.

[45] Noble E.E., Olson C.A., Davis E., Tsan L., Chen Y.-W., Schade R., Liu C., Suarez A., Jones R.B., de La Serre C. (2021). Gut Microbial Taxa Elevated by Dietary Sugar Disrupt Memory Function. Transl. Psychiatry.

[46] Guo X., Park Y., Freedman N.D., Sinha R., Hollenbeck A.R., Blair A., Chen H. (2014). Sweetened Beverages, Coffee, and Tea and Depression Risk among Older US Adults. PLoS ONE.

[47] Xia Y., Wang N., Yu B., Zhang Q., Liu L., Meng G., Wu H., Du H., Shi H., Guo X. (2017). Dietary Patterns Are Associated with Depressive Symptoms among Chinese Adults: A Case–Control Study with Propensity Score Matching. Eur. J. Nutr..

[48] Zahedi H., Kelishadi R., Heshmat R., Motlagh M.E., Ranjbar S.H., Ardalan G., Payab M., Chinian M., Asayesh H., Larijani B. (2014). Association between Junk Food Consumption and Mental Health in a National Sample of Iranian Children and Adolescents: The CASPIAN-IV Study. Nutrition.

[49] Palan S., Schitter C. (2018). Prolific.ac—A Subject Pool for Online Experiments. J. Behav. Exp. Financ..

[50] Kessler R.C., Berglund P., Demler O., Jin R., Koretz D., Merikangas K.R., Rush A.J., Walters E.E., Wang P.S. (2003). The epidemiology of major depressive disorder: Results from the National Comorbidity Survey Replication (NCS-R). JAMA.

[51] Illig T., Gieger C., Zhai G., Römisch-Margl W., Wang-Sattler R., Prehn C., Altmaier E., Kastenmüller G., Kato B.S., Mewes H.-W. (2010). A Genome-Wide Perspective of Genetic Variation in Human Metabolism. Nat. Genet..

